# Supplementary material for: Logical rotation of non-separable states via uniformly self-assembled chiral superstructures
Source: Nat Commun. 2024 Feb 6;15:1108. doi: 10.1038/s41467-024-45299-8 (PMC10847456; doi:10.1038/s41467-024-45299-8)
Supplement: Supplementary file 1 — Supplementary Information [file 41467_2024_45299_MOESM1_ESM.pdf]

Supplementary Information for

Logical rotation of non-separable states via uniformly self-assembled chiral superstructures

Yi-Heng Zhang[1], Si-Jia Liu[1], Peng Chen[1]*, Dong Zhu[1], Wen Chen[1], Shi-Jun Ge[1], Yu Wang[1], Zhi-Feng Zhang[1], and Yan-Qing Lu[1]*

[1]National Laboratory of Solid State Microstructures, Key Laboratory of Intelligent Optical Sensing and Manipulation, College of Engineering and Applied Sciences, and Collaborative Innovation Center of Advanced Microstructures, Nanjing University, Nanjing 210093, China.
*E-mail: chenpeng@nju.edu.cn, yqlu@nju.edu.cn

This Supplementary Information contains six Supplementary Notes, sixteen Supplementary Figures, and thirty-five References.

Supplementary Note 1: Vectorial non-separable states

Structured light possesses various degrees of freedom (DoFs), such as polarization, spatial mode, and time[1,2]. In classical optics, the non-separability can be achieved between multiple intrinsic DoFs of the light field[3-6]. The expression of a non-separable state cannot be factorized as a product of two or more independent terms. One way to construct non-separable states is to select some orthogonal states of the related DoFs, apply their tensor products as basis states, and make linear composition.

The vector beam (VB)[7,8], a typical kind of non-separable states, is featured by spatially-variant linear polarizations, and is non-separable between the polarization (i.e., spin) and the spatial (i.e., intrinsic orbital) DoFs. Here, we select the spin angular momentum (SAM) eigenstates $|-\sigma\rangle$ and $|+\sigma\rangle$, which denote the right- and left-handed circular polarization (RCP and LCP). While the orbital angular momentum (OAM) eigenstates[9,10] are selected as $|+l\rangle$ and $|-l\rangle$, featured by topological charge ±*l*. For photons, $\sigma = 1$, and we choose $l = 1$ without any loss of generality. We can make four tensor products as the candidates for basis vectors, namely $|-\sigma\rangle|+l\rangle$, $|+\sigma\rangle|-l\rangle$, $|-\sigma\rangle|-l\rangle$ and $|+\sigma\rangle|+l\rangle$. In total, six different combinations of basis vector pairs exist, but only two of them yield the non-separable form of VBs, mathematically expressed by

$$\begin{cases} |\psi_1\rangle = \cos\left(\dfrac{\Theta}{2}\right)\exp(i\Phi)|-\sigma\rangle|+l\rangle + \sin\left(\dfrac{\Theta}{2}\right)|+\sigma\rangle|-l\rangle \\ |\psi_2\rangle = \cos\left(\dfrac{\Theta}{2}\right)\exp(i\Phi)|-\sigma\rangle|-l\rangle + \sin\left(\dfrac{\Theta}{2}\right)|+\sigma\rangle|+l\rangle \end{cases}. \tag{S1}$$

The above expression of vectorial non-separable states has two parameters, Θ and Φ. Θ indicates the relative proportion of two basis vectors. When $\Theta = 0$ or π, the vectorial non-separable state reduces to the product of the SAM and the OAM eigenstates and the non-separability vanishes. Φ is the relative phase difference between two basis state.

As a geometric representation, the vectorial non-separable states can be mapped to the surface of the so-called higher-order Poincaré sphere (HOPS)[11], and each state corresponds to certain spherical coordinate (Θ, Φ). The HOPS of $|\psi_1\rangle$ with $l = +1$ is illustrated in Fig. S1a. Figure S1b displays $|\psi_1\rangle$ in the case of $l = -1$, which is equivalent to the $|\psi_2\rangle$ in equation (S1). In principle, the OAM value *l* can be any integer, so the HOPS is not limited to $l = \pm 1$, and the case of $l = +4$ is depicted in Fig. S1c. The vectorial non-separable state is featured by the donut-shaped intensity profile of radius $r \propto \sqrt{|l|}$, and its local polarization orientation rotates $|2\pi l|$ per circulation about the central singularity[1,7]. Note that when $l = 0$, the HOPS reduces to the common Poincaré sphere, and the non-separable state reduces to the homogeneous spin cebit (i.e., pure polarization), as involved in Fig. 4,S12 and Supplementary Note 6.

When $\Theta = \pi/2$ and Φ alters, the vectorial non-separable state evolves around the equator of the HOPS, namely the logical rotation around the *z*-axis. Visually, the local polarization directions of the VB rotate simultaneously and transform to another polarization distribution, as displayed in Fig. S1. In order to implement the proposed logical rotation, the relative phase shift between two basis states should be certain values, while other terms should keep constant.

Supplementary Note 2: Analytical theory of chiral liquid crystal optics at oblique incidence

Chiral liquid crystals (CLCs) are self-assembled periodic structures, where anisotropic LC molecules organize into uniform standing helices, namely the planar texture. According to the locally uniaxial model, the relative dielectric tensor of CLC can be written in the laboratory coordinate system as[12,13]

$$\varepsilon(z)=\begin{bmatrix}\varepsilon_{xx} & \varepsilon_{xy} & \varepsilon_{xz}\\ \varepsilon_{yx} & \varepsilon_{yy} & \varepsilon_{yz}\\ \varepsilon_{zx} & \varepsilon_{zy} & \varepsilon_{zz}\end{bmatrix}=\begin{bmatrix}\varepsilon_{\mathrm{m}}+\varepsilon_{\mathrm{m}}\delta\cos(4\pi z/p) & \varepsilon_{\mathrm{m}}\delta\sin(4\pi z/p) & 0\\ \varepsilon_{\mathrm{m}}\delta\sin(4\pi z/p) & \varepsilon_{\mathrm{m}}-\varepsilon_{\mathrm{m}}\delta\cos(4\pi z/p) & 0\\ 0 & 0 & \varepsilon_3\end{bmatrix}, \quad \text{(S2)}$$

where p is the helical pitch, $\varepsilon_{\mathrm{m}} = (\varepsilon_1 + \varepsilon_2) / 2$, and $\delta = (\varepsilon_1 - \varepsilon_2) / (\varepsilon_1 + \varepsilon_2)$. $\varepsilon_1 = n_{\mathrm{e}}^2$ and $\varepsilon_2 = \varepsilon_3 = n_{\mathrm{o}}^2$ are the three principal values, where $n_{\mathrm{o}}/n_{\mathrm{e}}$ is the ordinary/extraordinary refractive index of CLC, respectively. The helical axis is along the z-axis, and the local LC director at the surface is parallel to the x-axis. Owing to the complete translational symmetry along any direction perpendicular to the z-axis, the optical modes, namely the solutions to Maxwell equations, are given by the following form[14,15]:

$$\begin{bmatrix}\mathbf{E}\\ \mathbf{H}\end{bmatrix}=\begin{bmatrix}\mathbf{E}(z)\\ \mathbf{H}(z)\end{bmatrix}\exp(i\kappa x-i\omega t)\cdot \quad \text{(S3)}$$

Here, κ is the component (perpendicular to the helical axis) of the incident wave vector $\mathbf{k}$. $\kappa = |\mathbf{k}|\sin\theta = n\omega\sin\theta/c$, where θ is the incident angle, and n is the refractive index of the external media. Combining Maxwell equations with Eq. (S2) and (S3), the optical modes in the CLC are determined by the following wave equations[14,15]:

$$\begin{cases}\left(\dfrac{1}{\varepsilon_{\mathrm{m}}}\dfrac{d^2}{dz^2}+\dfrac{n^2\omega^2}{c^2}-\dfrac{\kappa^2}{\varepsilon_3}\right)E_x+\delta\left(\dfrac{n^2\omega^2}{c^2}-\dfrac{\kappa^2}{\varepsilon_3}\right)\left(E_x\cos\dfrac{4\pi z}{p}+E_y\sin\dfrac{4\pi z}{p}\right)=0\\ \left(\dfrac{1}{\varepsilon_{\mathrm{m}}}\dfrac{d^2}{dz^2}+\dfrac{n^2\omega^2}{c^2}-\dfrac{\kappa^2}{\varepsilon_{\mathrm{m}}}\right)E_y+\dfrac{n^2\omega^2}{c^2}\delta\left(E_x\sin\dfrac{4\pi z}{p}-E_y\cos\dfrac{4\pi z}{p}\right)=0\end{cases}. \quad \text{(S4)}$$

Equation (S4) is a coupled second-order differential equation system characterized by periodic coefficients. Once E_x and E_y are solved, the other components (i.e., E_z, H_x, H_y, H_z) can be calculated by Maxwell equations. Following Floquet-Lyapunov-Bloch theorem, the unknowns are further expanded to Fourier series:

$$\begin{bmatrix}E_x\\ E_y\end{bmatrix}=\frac{1}{2}\exp(i\kappa x-i\omega t)\sum_{m=-\infty}^{+\infty}\begin{bmatrix}u_m+v_m\\ iu_m-iv_m\end{bmatrix}\exp\left(i\beta z+i\frac{4\pi m}{p}z\right), \quad \text{(S5)}$$

where β is the component (parallel to the helical axis) of the wave vector $\mathbf{k}$. Substituting Eq. (S5) into Eq. (S4), we obtain a set of algebraic equations involving u_m and v_m, by equating the coefficients of each harmonic component. Following the standard scheme to solve linear equation systems, the rigorous solution of optical eigenmodes in the CLC can be attained, despite complicated mathematical process.

In practical problems, the description of the optical properties of the CLC sample requires us to know the specific combination of eigenmodes satisfying the boundary conditions[13]. With the solutions of the boundary problem, we can make theoretical predictions for given CLC structures and given incident conditions. For example, the light reflection and transmission in a CLC slab of finite thickness can be solved based on the continuity of tangential components of electric and magnetic fields at the front and the rear surfaces.

For normal incidence, $\kappa = 0$, and Eq. (S4) is simplified significantly. The rigorous solutions become practical because only several terms in the Fourier series of Eq. (S5) are nonzero[13]. All solutions can be written explicitly without infinite series. Given the uniform CLC structures and the incident polarization, the polarization properties of the transmitted light are derived as analytical functions of the wavelength, as plotted in Fig. 2b. More detailed mathematical process to obtain the dispersion curve of the optical rotatory power can be found

in Ref. [16,17]. Polarization figure of merit (PFM) can also be calculated to evaluate the polarization of the output state. PFM = 1 indicates ideal linear polarization, while PFM = 0 indicates ideal circular polarization (i.e., SAM eigenstates).

To investigate the optical properties of CLC at oblique incidence in an easier way, much effort has been devoted to reasonable approximations. One idea is to extend the conclusions for normal incidence to oblique incidence, by referring to the X-ray diffraction theory in crystal[12]. The periodic structures of CLC introduce the reciprocal vector along the helix axis. The incident wave vector, the reciprocal vector and the resultant wave vector obey the kinematic relation. At the incident angle θ, the component (parallel to the reciprocal vector) of incident wave vector **k** becomes $|\mathbf{k}|\cos\theta$, while, a normal incident wave vector **k**' with $|\mathbf{k}'| = |\mathbf{k}|\cos\theta$ has the same parallel component. Thus, intuitively speaking, the oblique incidence at θ resembles the normal incidence with a longer effective wavelength $\lambda_{\mathrm{eff}} = \lambda / \cos\theta$. This simple physical picture provides us some proper intuitions, especially about the blue shift of the first-order photonic band gap (PBG) at oblique incidence[12]. Nevertheless, it fails to supply convincing interpretations on the optical polarization properties.

To study more intriguing phenomena such as optical rotation and higher-order reflection, various perturbation methods[13,18] have been adopted to obtain approximate solutions of Eq. (S4). Following Sterling and Hayes's theory[18], we plot the dependence of optical rotatory power on the incident angle of CLC in Fig. 2c. This approximate relation implies that when the operating wavelength lies in the short-wavelength edge vicinity of PBG, the rotation of input polarization direction can be flexibly tuned by the incident angle. However, for comprehensive and quantitative knowledge of the optical properties in CLC, numerical technique will be a better choice.

Supplementary Note 3: Berreman's 4 × 4 matrix numerical algorithm

Berreman and Scheffer introduce a 4 × 4 matrix numerical technique to compute the light in any stratified and anisotropic media at normal or oblique incidence[19]. The dielectric tensor ε of CLC is expressed by Eq. (S2) and the light wave holds the form in Eq. (S3). Maxwell equations are organized as the following first-order differential equation system, expressed by a 4 × 4 matrix:

$$\frac{\partial}{\partial z}\begin{bmatrix} E_x \\ iH_y \\ E_y \\ -iH_x \end{bmatrix} = \frac{\omega}{c}\begin{bmatrix} -i\frac{\varepsilon_{xz}}{\varepsilon_{zz}}\sin\theta^* & 1-\frac{1}{\varepsilon_{zz}}\sin^2\theta^* & -i\frac{\varepsilon_{yz}}{\varepsilon_{zz}}\sin\theta^* & 0 \\ -\varepsilon_{xx}+\frac{\varepsilon_{xz}^2}{\varepsilon_{zz}} & -i\frac{\varepsilon_{xz}}{\varepsilon_{zz}}\sin\theta^* & \frac{\varepsilon_{xz}\varepsilon_{yz}}{\varepsilon_{zz}}-\varepsilon_{xy} & 0 \\ 0 & 0 & 0 & 1 \\ \frac{\varepsilon_{xz}\varepsilon_{yz}}{\varepsilon_{zz}}-\varepsilon_{xy} & -i\frac{\varepsilon_{yz}}{\varepsilon_{zz}}\sin\theta^* & -\varepsilon_{yy}+\frac{\varepsilon_{yz}^2}{\varepsilon_{zz}}+\sin^2\theta^* & 0 \end{bmatrix}\begin{bmatrix} E_x \\ iH_y \\ E_y \\ -iH_x \end{bmatrix}, \quad \text{(S6)}$$

or $\frac{\partial}{\partial z}\mathbf{f}(z) = \frac{\omega}{c}\mathbf{D}(z)\mathbf{f}(z)$.

Here, θ^* is the angle between the helical axis (i.e., the z-axis) and the beam propagation direction in the CLC sample. The CLC is treated as the cascade of homogeneous micro-layers of thickness Δz. The differential matrix $\mathbf{D}(z)$ does not vary appreciably over the small interval Δz (i.e., $\mathbf{D}(z)$ keeps approximately constant over $z \sim z + \Delta z$). So the differential equation Eq. (S6) can be integrated to a matrix equation Eq. (S7), which relates the light fields at the input surface and the output surface of the micro-layer.

$$\mathbf{f}(z+\Delta z) = \mathbf{P}(z)\mathbf{f}(z) = \exp\left[\frac{\omega \Delta z}{c}\mathbf{D}(z)\right]\mathbf{f}(z), \tag{S7}$$

where $\mathbf{P}(z)$ is the propogation matrix of the micro-layer between z and $z + \Delta z$. The results for larger propogation length can be obtained by multiplying the matrices in sequence. The shortest period in the CLC is half of the pitch, $p/2$. Suppose $p/2 = n\Delta z$ and the total thickness of CLC is $L = Np/2$, we can establish the relation between the light fields at the front surface ($z = 0$) and the rear surface ($z = L$) as following:

$$\mathbf{f}(L) = \left[\prod_{j=0}^{n-1}\mathbf{P}(j\Delta z)\right]^{N}\mathbf{f}(0) = \mathbf{F}\mathbf{f}(0). \tag{S8}$$

Here, $\mathbf{f}(0)$ describes the sum of the incident and the reflected light, while $\mathbf{f}(L)$ describes the transmitted light.

In experiments, it is more practicable to define θ as the angle between the incident light direction and the central normal axis of the device. The beam propagates through the glass substrate of the device and subsequently reaches the surface of CLC structures. The refraction at the air-glass interface obeys Snell's law, so the θ^* in Eq. (S6) is given by $\sin\theta = n_{\text{glass}}\sin\theta^*$. Using Eq. (S8) and the Maxwell equations in the external isotropic media, all information about the transmitted light and the reflected light can be derived from the incident light. By Berreman's 4 × 4 matrix algorithm, the homogeneous transmitted state $|\mathrm{E}_{\text{out}}\rangle$ corresponding to certain wavelength, incident angle and input polarization can be calculated (Fig. 4,S2,S6,S13-S15). Applying mesh grid method, we can further generalized the 4 × 4 matrix algorithm to deal with the inhomogeneous structured fields, and the simulated profiles are displayed in Fig. 5,6,S8,S14-S16.

The matrix $\mathbf{F}$ in Eq. (S8) summarizes the effects of the CLC sample on the incident light. Eigendecomposition of $\mathbf{F}$ will reveal the properties of optical eigenmodes in CLC, formulated as

$$\mathbf{F} = \sum_{j=1}^{4}\exp(ig_j)\mathbf{g}_j\mathbf{g}_j^T, \tag{S9}$$

where g_j represents the propogation constant of the j-th mode, and the polarization is expressed by the eigenvector $\mathbf{g}_j$ (i.e., the relations of E_x, E_y, H_x and H_y). Outside the PBG, we are only interested in the two forward-propagating modes, since the other two are back-propogating modes from the rear surface with similar form. As shown in Fig. S3, the polarizations of eigenmodes are generally LCP and RCP for a wide range of the incident angle, satisfying the requirement of the proposed operator $\mathbf{O}_{\text{spin}}$.

Supplementary Note 4: Comparison of the ideal and the chiral-superstructure-mediated logical rotation gates

For comparison, the ideal logical rotation operator in Eq. (2) is re-written as (normalization constants are omitted)

$$\mathbf{O}_{\text{spin}} \otimes \mathbf{I}_{\text{orb}} = \left[\exp(i\Phi)\left|-\sigma\right\rangle\left\langle-\sigma\right| + \left|+\sigma\right\rangle\left\langle+\sigma\right|\right] \otimes \left(\left|+l\right\rangle\left\langle+l\right| + \left|-l\right\rangle\left\langle-l\right|\right). \tag{S10}$$

The expression indicates three elementary requirements of the ideal logical rotation gate. First, the eigenstates of $\mathbf{O}_{\text{spin}}$ are just the SAM eigenstates. Second, there exists relative phase shift between the SAM eigenstates and Φ equals the logical rotation angle. Third, there is no influence on the orbital DoF.

For physical implementation of the proposed logical rotation gate, we applied the uniform left-handed chiral superstructures with sufficient interaction length, and the operating wavelength is within the short-wavelength edge vicinity of the PBG. Numerical results in Fig. S3 show that the polarizations of eigenmodes in CLC generally coincide with the SAM eigenstates, which is further proved by the measured output states with nearly linear polarization (Fig. 3-5). Theories and experiments (Fig. 2-6,S6,S8) collaborate to show that the rotation of local polarization direction significantly depends on the incident angle at the CLC device. The chiral superstructures are uniform without any appreciable influence on the orbital DoF. Thus, the CLC logical rotation gate is formulated as

$$\mathbf{M}_{\text{CLC}} = \left[\exp(i\Gamma_1)\left|\alpha_1\right\rangle\left\langle\alpha_1\right| + \exp(i\Gamma_2)\left|\alpha_2\right\rangle\left\langle\alpha_2\right|\right] \otimes \left(\left|+l\right\rangle\left\langle+l\right| + \left|-l\right\rangle\left\langle-l\right|\right). \tag{S11}$$

Here, $\left|\alpha_1\right\rangle$ and $\left|\alpha_2\right\rangle$ are the eigenstate vectors, while Γ_1 and Γ_2 are the corresponding dynamic phases accumulated in propagation. In left-handed CLC chiral superstructures, $\left|\alpha_1\right\rangle \approx \left|-\sigma\right\rangle$ and $\left|\alpha_2\right\rangle \approx \left|+\sigma\right\rangle$ [13,20]. When the operating wavelength lies in the short-wavelength edge vicinity, the phase difference Γ_1 - Γ_2 is positive and regulated by the incident angle. Equation (S11) agrees with Eq. (S10), indicating that the CLC device approximately behaves as the ideal logical rotation gate. While in right-handed CLCs, $\left|\alpha_1\right\rangle \approx \left|+\sigma\right\rangle$ and $\left|\alpha_2\right\rangle \approx \left|-\sigma\right\rangle$ [13,20], so the direction of the polarization rotation is opposite to that of left-handed ones, as displayed in Fig. S15.

When the incident angle grows to be large enough, the PBG will blue shift and finally cover the operating wavelength. As shown in Fig. 5f, the SAM eigenstates are separated into the reflection and the transmission channel. Distinct from the logical rotation operator described by Eq. (S11), the left-handed CLC device executes spin-selective projection in both channels, which is mathematically expressed by

$$\begin{cases} \mathbf{T} = \left(\left|-\sigma\right\rangle\left\langle-\sigma\right|\right) \otimes \left(\left|+l\right\rangle\left\langle+l\right| + \left|-l\right\rangle\left\langle-l\right|\right) \\ \mathbf{R} = \left(\left|+\sigma\right\rangle\left\langle+\sigma\right|\right) \otimes \left(\left|-l\right\rangle\left\langle+l\right| + \left|+l\right\rangle\left\langle-l\right|\right) \end{cases}. \tag{S12}$$

The normalization constants and global phases are omitted here. **T** describes the operation in the transmission channel, where RCP is selectively transmitted. **R** represents the selective reflection of LCP. The OAM is reversed due to the light reflection, while the spin is conserved in the local coordinate system, because the spin-selective reflection in CLC is essentially the coupling between counter-propagating light waves instead of Fresnel reflection at the surface[13].

Supplementary Note 5: Logical rotation controlled by the photonic wave vector

The logical rotation of spin cebits and vectorial non-separable states depends on the incident angle θ at the CLC device. In fact, the incident angle is determined by both the beam and the device, formulated as $\theta = \theta_{mech} - \theta_{phot}$, where θ_{mech} is the tilt angle of the CLC device and θ_{phot} is the wave vector direction of incident light. Thus, the controllable logical rotation can be implemented in two control modes (Fig. S10). The *mechanical mode* is to keep $\theta_{phot} = 0°$ and to alter θ_{mech} by mechanically rotating the CLC device. While the other mode, named as *wave vector mode*, is to keep θ_{mech} constant and alter the wave vector direction θ_{phot}. Note that θ_{phot} can be controlled passively by optical elements like gratings.

The control mode presented in Fig. 5,S8 is the *mechanical mode*. One non-separable cebit is accepted as an input, and an output cebit is determined by the external physical quantity θ_{mech}. Mechanical rotation of the CLC device is the only required operation to dynamically perform a variety of distinguished logic gates. In this mode, the CLC logic gate is analogous to the classical NOT gate and the qubit rotation (including Pauli-Z gate, S gate et al)[21], with an additional parameter θ_{mech} for controllability (Fig. S10). A connection between the mechanical state (tilt angle) and the photonic non-separable state is established, which enables angular motion tracking (Fig. 6b,c).

In contrast, the control in *wave vector mode* is the photonic wave vector (i.e., θ_{phot}). Distinguished from the tilt angle θ_{mech}, the wave vector is an intrinsic DoF of light. When the logical rotation is executed, the wave vector DoF acts as the control, and the non-separable state of the spin and the orbital DoFs serve as the target, as illustrated in Fig. 6d. The CLC device in *wave vector mode* is analogous to two-bit gates for Boolean circuits and CNOT gates for quantum networks[21], since both the control and the target are carried by the photonic state (Fig. S10). Such control mode would pave the way for a logic network of non-separable states. The discussion on this issue is presented later in Supplementary Note 6.

To experimentally demonstrate the logical rotation controlled by the photonic wave vector, we design the quantum-like circuit in Fig. S11a, and physically implement it with the setup in Fig. 6e. By employing a combination of a *q*-plate and a 1 × 4 Dammann grating, we generate the same initial non-separable state with radial polarization, but encoded with different θ_{phot} in four channels. The 1 × 4 Dammann grating is a binary-phase (0 and π) grating composed of a set of normalized phase transition points, selected as {0, 0.22057, 0.44563, 0.5, 0.72057, 0.94563, 1}[22]. The grating period is Λ_{DG} = 46.5 μm. The normal incident beam is diffracted into four orders with nearly equal-energy distribution, whose directions obey the following grating equations:

$$\begin{cases} \theta_{phot} = \arcsin\left(-3\lambda/\Lambda_{DG}\right) & \text{in Channel 1} \\ \theta_{phot} = \arcsin\left(-\lambda/\Lambda_{DG}\right) & \text{in Channel 2} \\ \theta_{phot} = \arcsin\left(+\lambda/\Lambda_{DG}\right) & \text{in Channel 3} \\ \theta_{phot} = \arcsin\left(+3\lambda/\Lambda_{DG}\right) & \text{in Channel 4} \end{cases}, \tag{S13}$$

where λ = 632.8 nm. As a result, the VBs in four channels propagate along the direction of θ_{phot} = -2.34°, -0.78°, +0.78°, and +2.34°, respectively. Afterwards, the non-separable states in four channels are simultaneously sent to the same CLC logic gate tilted at fixed θ_{mech} = 17.84°, and the resultant incident angles are $\theta = \theta_{mech} - \theta_{phot}$ = 20.18°, 18.62°, 17.06°, and 15.50°, respectively. As displayed in Fig. 6f and summarized in Fig. S11b,c, the analyzed intensity profiles with two separated lobes exhibit different orientations, implying different output VB states in four channels. Since the input non-separable states are the same, the logical rotation is efficiently controlled by the photonic wave vector, showing parallel and massive processing manner.

Although the logical rotation is controlled by altering θ_{phot}, the constant θ_{mech} also plays an important role in

such *wave vector mode*. The value of θ mainly depends on θ_{mech}, because θ_{phot} is usually relatively small compared to θ_{mech}. In the proposed case (Fig. 6,S11), θ_{mech} = 17.84°, and the overall varying θ is located in the interval from 15.50° to 20.18°. The preset θ_{mech} determines the operating region of the CLC device, analogous to the biasing of the electronic transistor to fix the quiescent point at the desired location[23]. A different θ_{mech} would lead to a different operating region, as well as different dependence of Φ on θ_{phot}. This offers a practical way to endow the CLC logic gate with diverse wave-vector-controllability.

Supplementary Note 6: The logic network of non-separable states and its demo for spin cebits

A step from the single logical rotation gate towards the complete logic network is vital for developing algorithms or protocols based on non-separable states. A logic network is essentially a logic control system where (i) the output state depends on the input state and on a control state following a certain law, and (ii) the output of a stage can be inserted into the input and the control of a successive stage[21,23]. For example, the combination of electronic logic gates[23], such as NOT gates, AND gates and OR gates, satisfies both requirements and assemble into a complete processor. Another example is the set of Clifford gates in quantum circuits[24], which includes single-qubit gates (Hadamard gate, S gate, and T gate) as well as the controlled gate (CNOT gate). Our discussion on the logic network of non-separable states is organized into four parts:

(i) applicability of the proposed CLC device in logic network;
(ii) designed configuration;
(iii) proof-of-principle demonstration;
(iv) potential applications of this proposed logic network.

(i) Applicability of the proposed CLC device in logic network

In *mechanical mode*, the input and the output are both photonic states, while the control is the tilt angle of the device, a mechanical quantity external to the light. The logical rotation controlled by tilt angle θ_{mech} should be understood more precisely as 'external-parameter dependence', slightly different from 'logic control'. The first requirement of logic network is satisfied, but the second is not. Similar to the fact that a complete logic network cannot be constructed only with single-qubit gates but without controlled gates[24], we cannot build a logic control system only with CLC logical rotation gates in this mode.

Fortunately, we can turn to *wave vector mode*. As expounded in Supplementary Note 5, the control of logical rotation is the wave vector direction, an intrinsic DoF of light, instead of an external quantity. The output non-separable state is determined by the input non-separable state and the wave vector (i.e., θ_{phot}), which meets with the first requirement of a logic network. The control manner of our CLC device is unidirectional. The wave vector DoF acts as the control, and the non-separable state in the spin and the orbital DoFs serves as the target. On one hand, the CLC logical rotation gate is similar to the CNOT gate on the aspect of controllability (Fig. S10). On the other hand, it is a major difference that the control and the target of a CLC gate belong to distinct DoFs but those of a CNOT gate are generally the quantum particles of the same kind[21,24]. Thus, the output of a

CNOT gate can directly act as the control of a successive one, while it is indirect for our CLC device. Fortunately, despite different DoFs, the control and the input/output are all embedded in the photonic state. Accordingly, a reverse-controlling unit can be proposed to modulate photonic wave vector (i.e., θ_{phot}) dependent on incident non-separable states, so that the output of a CLC logical rotation gate can be inserted into the control. By assigning a reverse-controlling unit between two CLC logical rotation gates, the output of the former stage is able to affect the input and the control of the next stage, so the second requirement of a logic network is satisfied as well.

(ii) Designed configuration

It is noteworthy that the photonic state with multiple DoFs should be understood as a package of multiple components. One component is the non-separable cebit associated with the spin and orbital DoFs, serving as the input and output of the logical rotation gate. The other component is the photonic wave vector DoF acting as the control. Diverse DoFs in VBs allow us to build a logic network of non-separable states using the CLC logical rotation gates coordinated with the reverse-controlling units. The designed configuration is illustrated in Fig. S12a. Non-separable state rotation gates and reverse-controlling units are assembled in an alternative sequence. The logical rotation of the input non-separable state is controlled by θ_{phot}, producing an output non-separable state. This output is fed to a reverse-controlling unit, and inserted into the wave vector DoF, acting as the control of the successive logical rotation gate. The logical rotation controlled by photonic wave vector has been well verified (see Supplementary Note 5 and Fig. 6,S11). In addition, thanks to the impressive progress in on-demand engineering of multiple DoFs of light, the suggested reverse-controlling unit is highly possible to be realized by carefully-designed metasurfaces or diffractive neural networks[25-27].

(iii) Proof-of-principle demonstration

As proof-of-principle, we design a simplified version of the logic network, which can be well demonstrated with easily-accessible optical elements. As shown in Fig. S12b, the basic structure of the logic network remains unchanged, while the non-separable state is reduced to the homogeneous polarization state (i.e., spin cebit, corresponding to $l = 0$). The experimental setup is illustrated in Fig. S12c. The operating wavelength is $\lambda = 632.8$ nm. Two CLC devices ($\theta_{\mathrm{mech}} = 16.55°$) are applied to clarify how the former stage affects the successive logical rotation. Here, the logical rotation of spin cebit is around the equator of the common Poincaré sphere (Fig. 4), quantified by the polarization orientation ϕ, and the logical rotation angle $\Phi = 2\phi$. The reverse-controlling unit for spin cebits is physically realized via the polarization grating sandwiched by two quarter-wave plates (QWPs). The polarization grating[28] can be understood as a half-wave plate with linearly varying orientation of local optical axes in the horizontal direction, formulated as $\alpha = -\pi x/\Lambda_{\mathrm{PG}}$. Here, $\Lambda_{\mathrm{PG}} = 27.3$ μm. Owing to the geometric phases, the incident beam with LCP/RCP will be deflected to the $-x/+x$ direction, and transformed into the opposite chirality[28]. The QWP before the polarization grating is applied to transform the linearly polarized state into a circularly polarized state, and the QWP after the grating converts the output beam back to the original linear polarization. The functions of the whole reverse-controlling unit can be summarized as following: (i) For horizontal polarization input ($\phi = 0°$), θ_{phot} is altered by $\sin\theta_{\mathrm{out}} = \sin\theta_{\mathrm{in}} - \lambda/\Lambda_{\mathrm{PG}}$, and the output cebit remains $|\phi = 0°\rangle$; (ii) For vertical polarization input ($\phi = 90°$), θ_{phot} is altered by $\sin\theta_{\mathrm{out}} = \sin\theta_{\mathrm{in}} + \lambda/\Lambda_{\mathrm{PG}}$, and the output cebit remains $|\phi = 90°\rangle$. Obviously, the variation in θ_{phot} (which can be the control of the next stage) is dependent on the incident spin cebit (the output state of the former stage).

In this network, all CLC logic gates are kept static without any mechanical rotation. Notably, we only prepare the spin cebit and the wave vector of the initial photonic state (step I), and all subsequent states (step II, III, IV)

evolve automatically by the aforementioned control rules. Three typical conditions of the initial state are performed, and the polarization states in the network are measured with a polarimeter and presented in Fig. S12d,e. Condition 1 and Condition 2 share the same initial spin cebit $|\phi = 0.0°\rangle$ (step I). Owing to different wave vector directions θ_{phot}, the output of the first CLC logical rotation gate (step II) is $|\phi = 0.2°\rangle$ for Condition 1 and $|\phi = 89.4°\rangle$ for Condition 2, respectively. The different output spin cebit leads to the negative/positive variation in θ_{phot} through the reverse-controlling unit (from step II to step III). Thus, the control of the second CLC logical rotation gate is modulated in response to the output of the first CLC gate. Comparison of Condition 2 and Condition 3 will further explain the function of the reverse-controlling unit. In the output of the first CLC logical rotation gate (step II), both photonic states share the same wave vector direction θ_{phot} = -2.55°, while their spin cebits are $|\phi = 89.4°\rangle$ and $|\phi = 0.0°\rangle$, respectively. Dependent on these spin cebits in step II, the reverse-controlling unit alters their wave vector and mainly keeps respective spin cebit, producing $|\theta_{\text{phot}} = -1.22°\rangle|\phi = 89.9°\rangle$ and $|\theta_{\text{phot}} = -3.88°\rangle|\phi = 0.4°\rangle$ in step III, respectively. After the second CLC logical rotation (step IV), the output spin cebits become $|\phi = 148.2°\rangle$ and $|\phi = 119.9°\rangle$, respectively. The polarization rotation from step III to IV for Condition 2 is $\Delta\phi$ = 148.2° - 89.9° = 58.3° (modulo 180°), corresponding to the logical rotation angle $\Phi = 0.65\pi$, which shows a clear contrast to $\Delta\phi$ = 119.9° - 0.4° = 119.5° (modulo 180°) and $\Phi = 1.33\pi$ for Condition 3.

In this proof-of-principle network, (i) the output spin cebit of the CLC logical rotation gate is determined by the input spin cebit and the wave vector, and (ii) the output spin cebit of the former stage is inserted into the wave vector via a reverse-controlling unit, acting as the control of the successive stage. Two requirements of a logic network are well fulfilled by our proposed configuration. Though only the simplified version for spin cebits is presented here, the logic network for vectorial non-separable states would be rationally expected, if a competent reverse-controlling unit is adopted. Generally speaking, a reverse-controlling unit needs to map a set of non-separable states to a set of propagating directions. This task involves sophisticated engineering of the spin, orbital, and wave vector DoFs of light. Recent researches in machine-learning inverse-design, meta-atom coupling models and innovative configurations have facilitated diverse forms of light-matter interactions in metasurfaces[29,30]. Featured by custom-designed manipulation of complex light field, especially those massive VBs[25-27], metasurfaces are very promising candidates for the reverse-controlling unit. Accordingly, the logic network for vectorial non-separable states could be established and carefully optimized for certain potential applications.

(iv) Potential applications of this proposed logic network

One potential application of this proposed logic network is the analogous version of quantum walks, which are instrumental computation tools for studying phenomena in condensed matters[31,32]. As an exemplary protocol, the lattice in common quantum walks would be encoded in the wave vector DoF, and the coin would be encoded into the non-separable state. Correspondingly, the coin rotation can be implemented by the CLC logical rotation gates, while the walker translation can be realized by the reverse-controlling units. These contribute to an analogous version of quantum walk based on classical non-separable states. When the non-separable state is reduced to the spin cebit (i.e., polarization), this elementary case can also be realized by a stack of wave plates and polarization gratings, and used to simulate a Chern insulator[31]. Compared to spin cebits, non-separable states are associated with both spin and orbital DoFs, so we can encode the coin into more complex form. In addition, the non-trivial rules of the logical rotation allow us to design the coin rotation in a highly flexible way. These might open new avenues for modeling complicated physical systems.

Moreover, in a more general view, the proposed logic network here is a kind of optical computing system

where parallel computation can be executed in the speed of light propagation[33]. A rich set of response functions can be rationally constructed from the non-standard logical rotation rules, which are determined by some specific factors including the pitch and the chirality of the CLC material and the preset tilt angle of the CLC device. Notably, a non-separable cebit offers a much larger state space than a traditional bit, and this would greatly extend the information capacity. In all, we anticipate that such a functional logic network, established on the CLC logical rotation gates and other versatile optical elements, would unveil new opportunities of non-separable states in high-dimensional photonics, optical computing and optical informatics.

Supplementary Figures

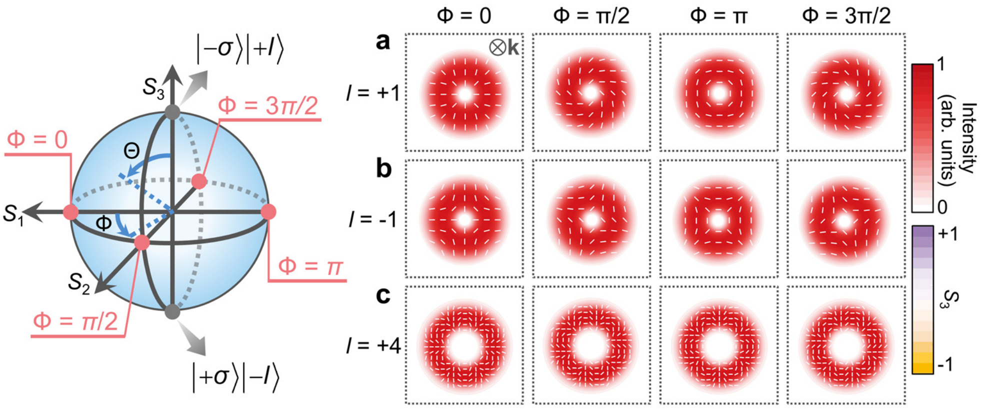

Fig. S1 | Schematic illustration of the logical space of vectorial non-separable states, namely the HOPS, spanned by the basis vectors of $|-\sigma\rangle|+l\rangle$ and $|+\sigma\rangle|-l\rangle$, with (**a**) $l = +1$, (**b**) $l = -1$, and (**c**) $l = +4$, respectively. (Θ, Φ) is the spherical coordinate. For certain points at the equator, polarization maps are shown as false-color intensity and polarization ellipses distribution, whose colors depend on Stokes parameter S_3. As the state logically rotates around the equator, the local polarization directions of the VB rotate simultaneously. All beam profiles were observed under the condition that the beam propagates away from the observer.

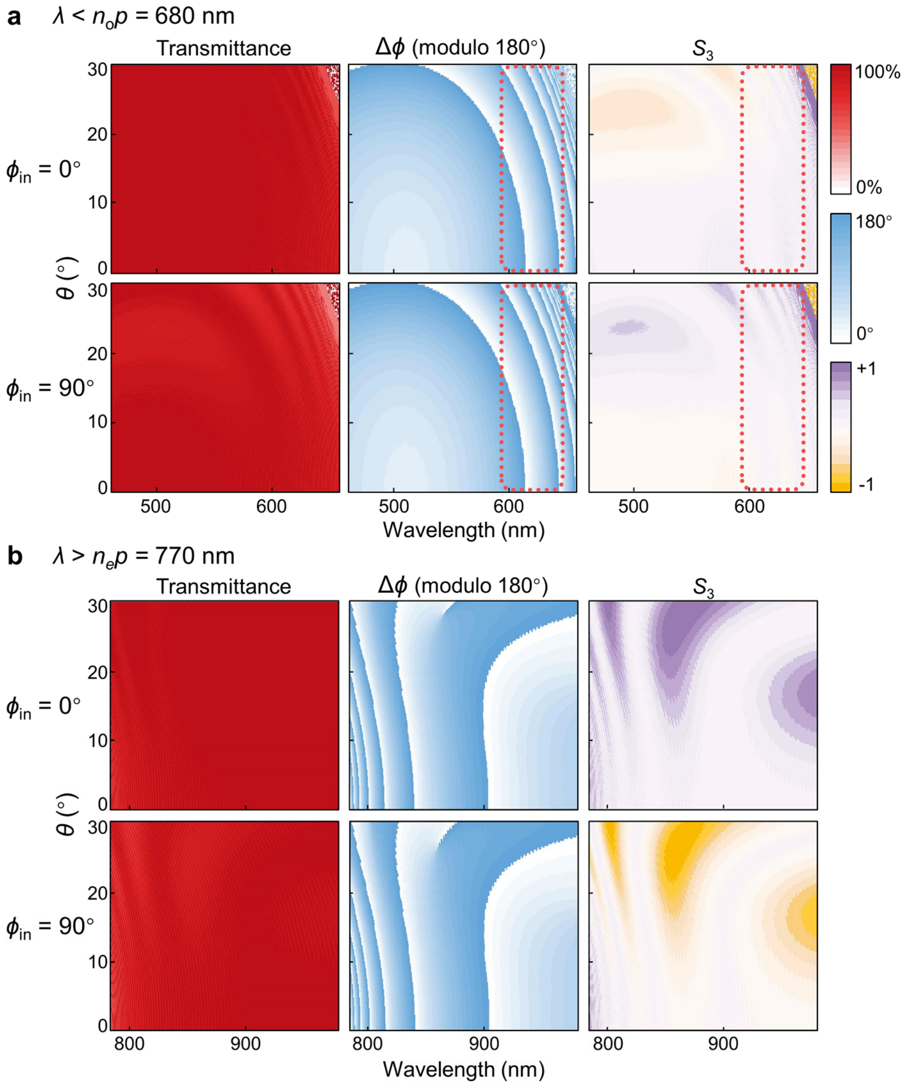

Fig. S2 | Simulated results of the transmittance without analyzer, polarization rotation $\Delta\phi$, and Stokes parameter S_3 as a function of the incident angle θ and the wavelength in the spectrum region with (**a**) shorter and (**b**) longer wavelengths than the PBG, respectively. The PBG is between 680 nm and 770 nm for normal incidence. The input state is horizontal (ϕ_{in} = 0°) and vertical (ϕ_{in} = 90°) linear polarization, respectively. The transmittance without analyzer keeps high for various wavelengths and incident angles. The polarization rotation is sensitive to θ for wavelengths in the vicinity of PBG. In the short-wavelength edge vicinity labelled by red dotted circles in (**a**), the output state maintains generally linearly polarized with PFM ≥ 0.85 over a wide range of incident angle. However, in the long-wavelength edge vicinity in (**b**), the PFM of the output state drops below 0.85 when θ grows over 15°, indicating a large deviation from the linear polarization. PFM is related to S_3 by $\text{PFM} = \sqrt{1-S_3^2}$.

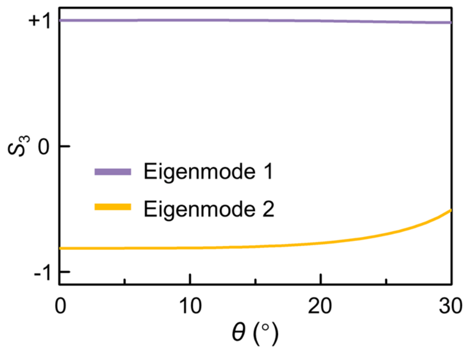

Fig. S3 | Simulated dependence of the Stokes parameter S_3 of the optical eigenmodes in CLC on the incident angle. The operating wavelength 632.8 nm lies within the short-wavelength edge vicinity of PBG. Over a wide range of θ, the polarizations of eigenmodes generally coincide with the SAM eigenstates, namely mode 1 for RCP and mode 2 for LCP.

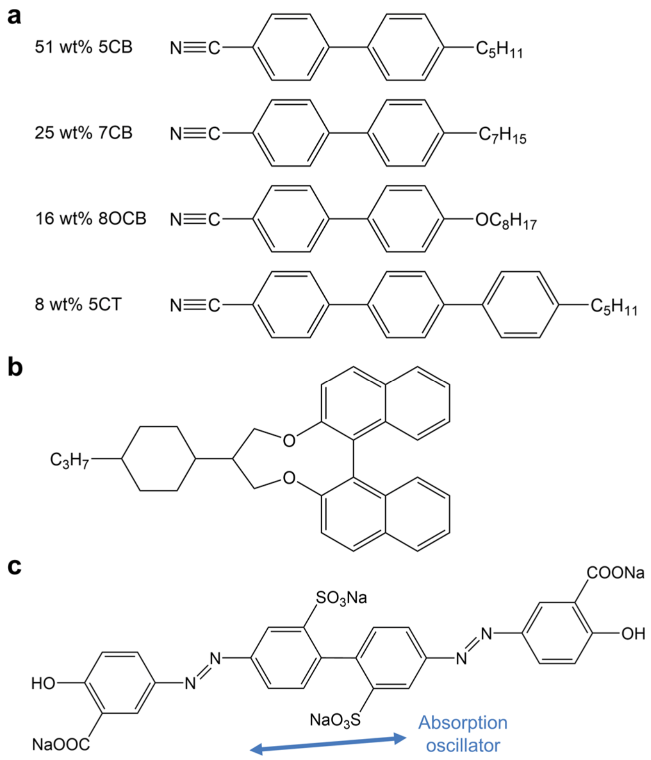

Fig. S4 | Molecule structures of (**a**) the nematic LC mixture E7, (**b**) the left-handed/right-handed chiral dopant S5011/R5011, and (**c**) the photoalignment agent SD1.

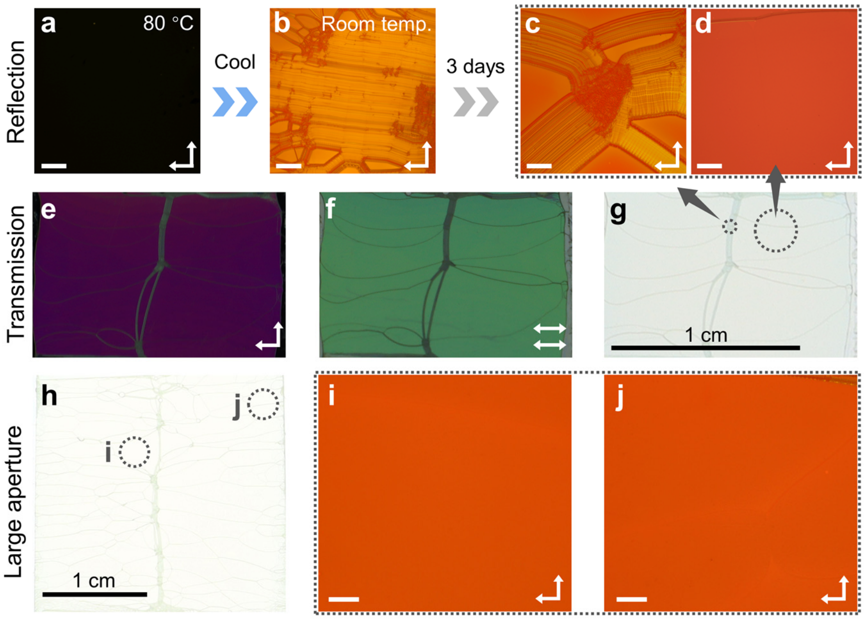

Fig. S5 | Fabrication and photographs of the uniform chiral superstructures. **a-d**, Reflective polarized micrographs of the CLC logic gate (**a**) at 80 °C, (**b**) cooled to room temperature, and (**c**, **d**) after kept in the dark for 3 days, respectively. The oily streaks (**b**), whose wall defect lines are mostly perpendicular to the unsealed boundaries, gradually merge and form uniform planar textures (**d**), owing to the system's tendency to minimize the free energy[20]. **e-g**, Macroscopic photographs of the CLC device sandwiched by (**e**) crossed polarizers, (**f**) parallel polarizers, and (**g**) no polarizer, respectively. The narrow ribbon-like region of oily streak defects (**c**) and the functional region of planar textures (**d**) are indicated in (**g**). **h-j**, Macroscopic photograph (**h**) and reflective polarized micrographs (**i**, **j**) of a large aperture CLC device with the functional region of about 5.8 cm^2. The aperture of the CLC device can be scaled up by using the substrates of sufficiently large size, which proves the scalability of the proposed self-assembly technique. The scale bars in micrographs and macroscopic photographs are 100 μm and 1 cm, respectively.

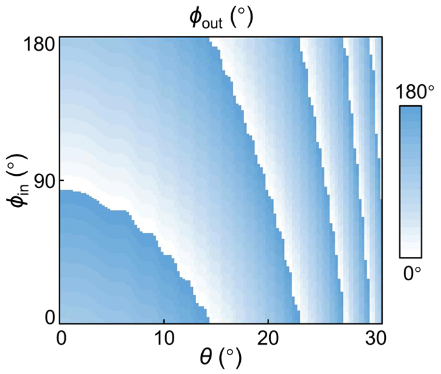

Fig. S6 | Simulated results of the output polarization direction ϕ_{out} as a function of the incident angle θ ranging from 0° to 30° and the input linear polarization direction ϕ_{in} ranging from 0° to 180°. The operating wavelength 632.8 nm lies within the short-wavelength edge vicinity of PBG.

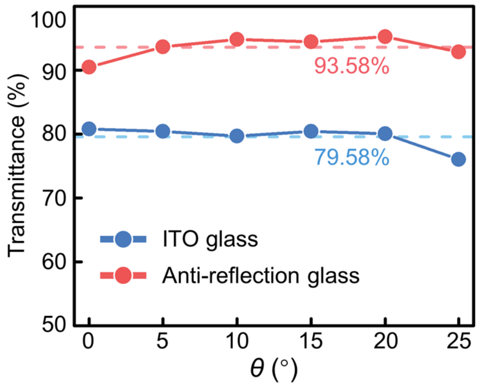

Fig. S7 | Transmittance of the CLC device under 632.8 nm laser illuminence at different incident angles. The substrates are common indium-tin-oxide (ITO) glass (blue), and glass with anti-reflection coating (red), respectively. The average values (horizontal dashed lines) are 79.58% and 93.58%, respectively.

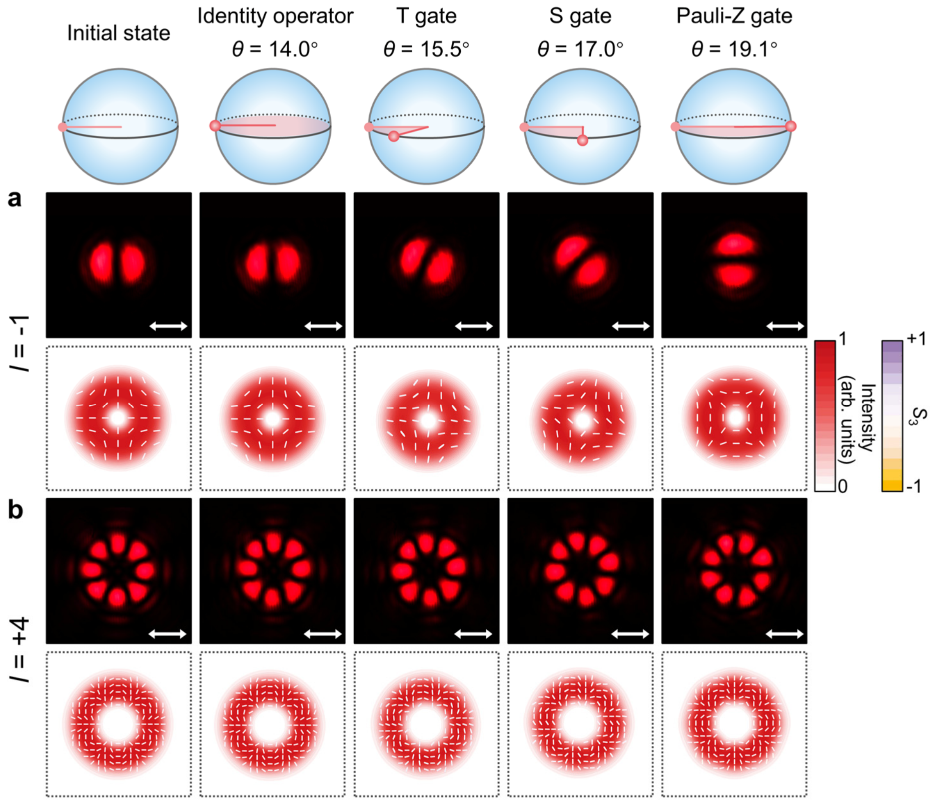

Fig. S8 | Logical rotation of the vectorial non-separable states at 632.8 nm with (**a**) l = -1, and (**b**) l = +4, respectively. Schematic illustrations of the state on the HOPS, captured analyzed intensity profiles, and simulated polarization maps of the initial state and different output states when the CLC device performs an identity operator, a T gate, an S gate, and a Pauli-Z gate, corresponding to θ = 14.0°, 15.5°, 17.0°, and 19.1°, respectively. White arrows denote horizontal analyzers. Polarization maps are shown as false-color intensity and polarization ellipses colored according to S_3. The analyzed intensity profiles contain $|2l|$ separated lobes. Since the polarization distribution of the non-separable states evolves during logical rotation, the orientations of intensity minima (i.e., dark stripes) rotate accordingly.

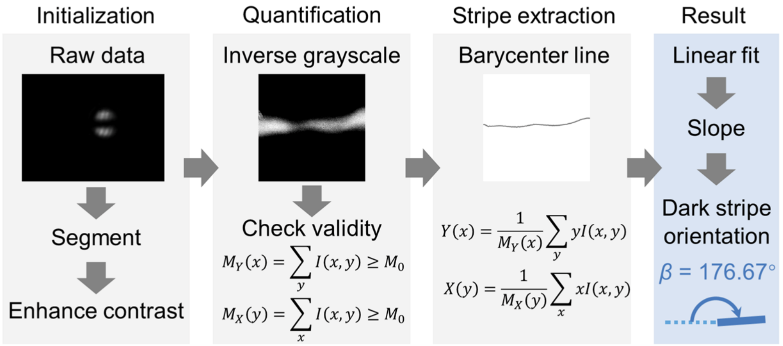

Fig. S9 | Flow chart of extracting the dark stripe orientation angle β from the analyzed intensity profile. The proposed algorithm is composed of four stages: Initialization to get an image of proper size and contrast; Quantification of the intensity distribution by inversing the grayscale and checking the stripe validity; Recognition of the stripe by the grayscale barycenter method; Final result from linear regression of the barycenter line.

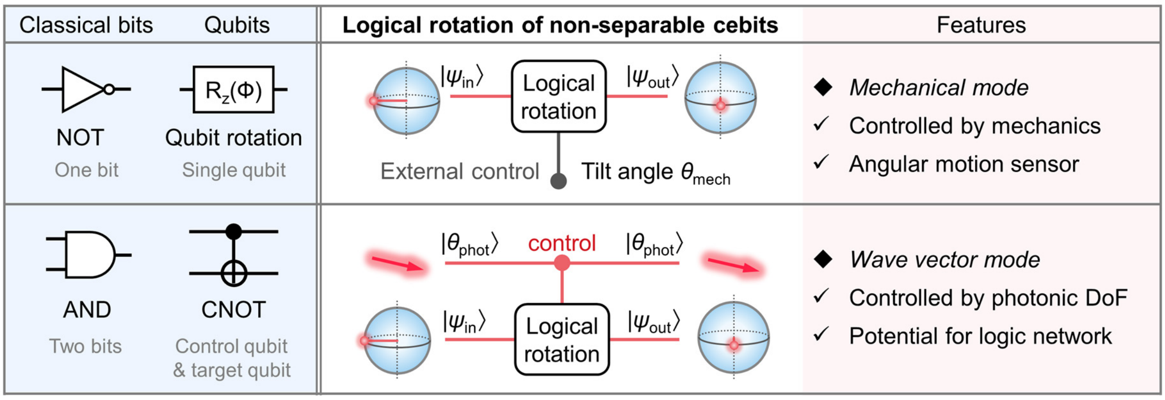

Fig. S10 | Two control modes of the logical rotation of non-separable states, and their analogues in classical and quantum circuits. The logical rotation angle Φ depends on the incident angle $\theta = \theta_{mech} - \theta_{phot}$, where θ_{mech} is the tilt angle of the CLC device and θ_{phot} is the wave vector direction of light. In *mechanical mode*, one non-separable cebit is accepted as an input, and an output cebit is determined by the external physical quantity θ_{mech}, analogous to the classical NOT gate and the qubit rotation (including Pauli-Z gate, S gate et al). In this mode, the device can also serve as a sensor for angular motion tracking. While in *wave vector mode*, the wave vector (i.e., θ_{phot}), an intrinsic DoF of light, controls the logical rotation of non-separable states. Similar to two-bit gates for Boolean circuits and CNOT gates for quantum networks, the CLC logical rotation gates can assemble into a logic network in principle, which is discussed in Supplementary Note 6.

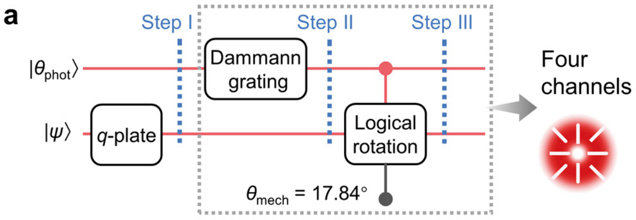

b

Step I	Step II	Step III
$|\theta_{phot} = 0°\rangle$ $|\Phi = 0\rangle$	$|\theta_{phot} = -2.34°\rangle$ $|\Phi = 0\rangle$	$|\theta_{phot} = -2.34°\rangle$ $|\Phi = 1.31\pi\rangle$
	$|\theta_{phot} = -0.78°\rangle$ $|\Phi = 0\rangle$	$|\theta_{phot} = -0.78°\rangle$ $|\Phi = 0.88\pi\rangle$
	$|\theta_{phot} = +0.78°\rangle$ $|\Phi = 0\rangle$	$|\theta_{phot} = +0.78°\rangle$ $|\Phi = 0.52\pi\rangle$
	$|\theta_{phot} = +2.34°\rangle$ $|\Phi = 0\rangle$	$|\theta_{phot} = +2.34°\rangle$ $|\Phi = 0.25\pi\rangle$

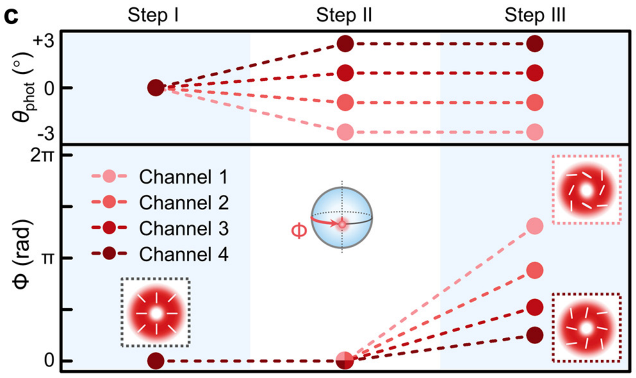

Fig. S11 | a, Circuit of the logical rotation controlled by wave vector. The upper horizontal line denotes the wave vector DoF, while the lower line denotes non-separable states. Two short vertical lines with round endpoints denote the control ports of the logical rotation gate, where θ_{mech} = 17.84° and θ_{phot} is different. Four channels are processed synchronously with the same tilted CLC device. Related experimental setup and results are included in Fig. 6e,f. The radially polarized VB $|\Phi=0\rangle$ is generated by the q-plate, and diffracted by the Dammann grating into four channels with θ_{phot} = -2.34°, -0.78°, +0.78°, and +2.34°, corresponding to $\theta = \theta_{mech} - \theta_{phot}$ = 20.18°, 18.62°, 17.06°, and 15.50°, respectively. (**b**) Table and (**c**) chart of the photonic state evolution in each channel. Inset: illustrations of the polarization distribution of the non-separable states in step I, and in Channel 1, 4 in step III, respectively. The logical rotation is well controlled by wave vector θ_{phot}.

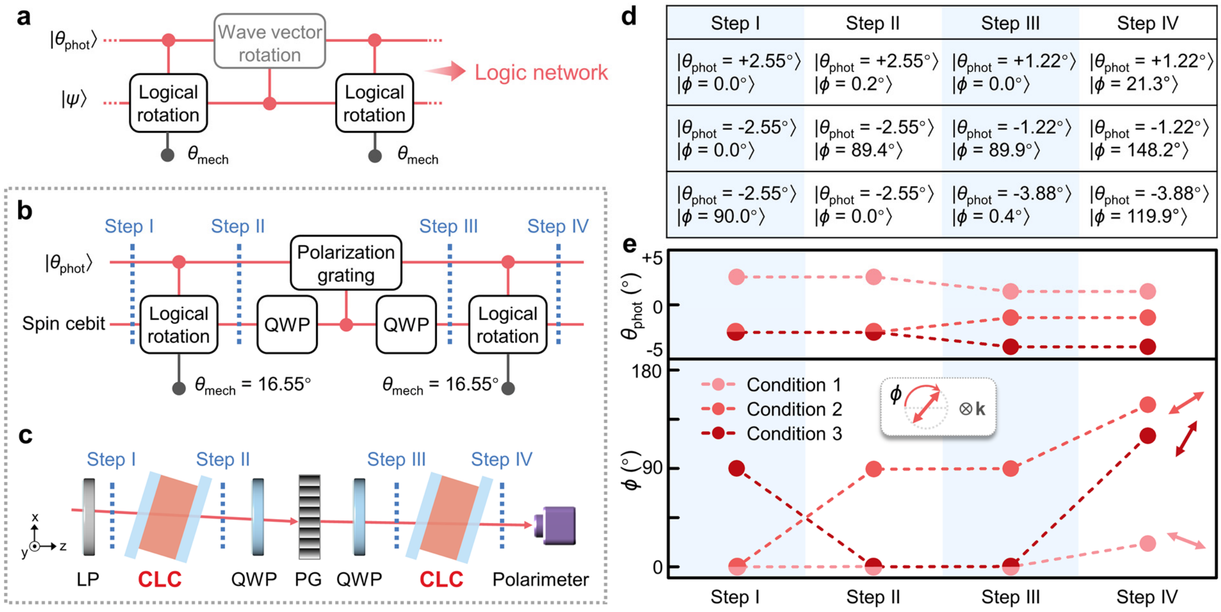

Fig. S12 | a, Circuit of a logic network. The upper/lower horizontal line denotes the wave vector DoF/the non-separable state, respectively. Short vertical lines with round endpoints denote the control ports. A certain constant is fixed to θ_{mech}, determining the operating region of the CLC logical rotation gate. Controlled by θ_{phot}, the CLC gate transforms the input non-separable state into the output state. This output is fed to a reverse-controlling unit, which can modulate θ_{phot} according to the non-separable state itself. In this way, the non-separable state in the former stage is inserted into the wave vector DoF (i.e., θ_{phot}), acting as the control of the successive logical rotation gate. CLC logical rotation gates together with reverse-controlling units can assemble into a complete logic network in principle. **b-c**, Circuit (**b**) and experimental optical setup (**c**) of the proof-of-principle logic network. Here, we consider the reduced non-separable state, i.e., the spin cebit (polarization). The reverse-controlling unit is implemented by the integration of two quarter-wave plates (QWPs) and a polarization grating (PG) of period Λ_{PG} = 27.3 μm. Incident horizontal/vertical polarization leads to a negative/positive variation in θ_{phot}. θ_{mech} is set as a constant of 16.55°. The spin cebit in each step is characterized by a polarimeter. **d-e**, Measured photonic state evolution table (**d**) and chart (**e**) of the proof-of-principle logic network. In three conditions, different initial states are prepared (step I). Comparing Condition 2 with Condition 1 or 3, it is obvious that the output ϕ of the first logical rotation gate (step II) affects the successive logical rotation (from step III to step IV). The final output polarization states in three conditions are illustrated as well.

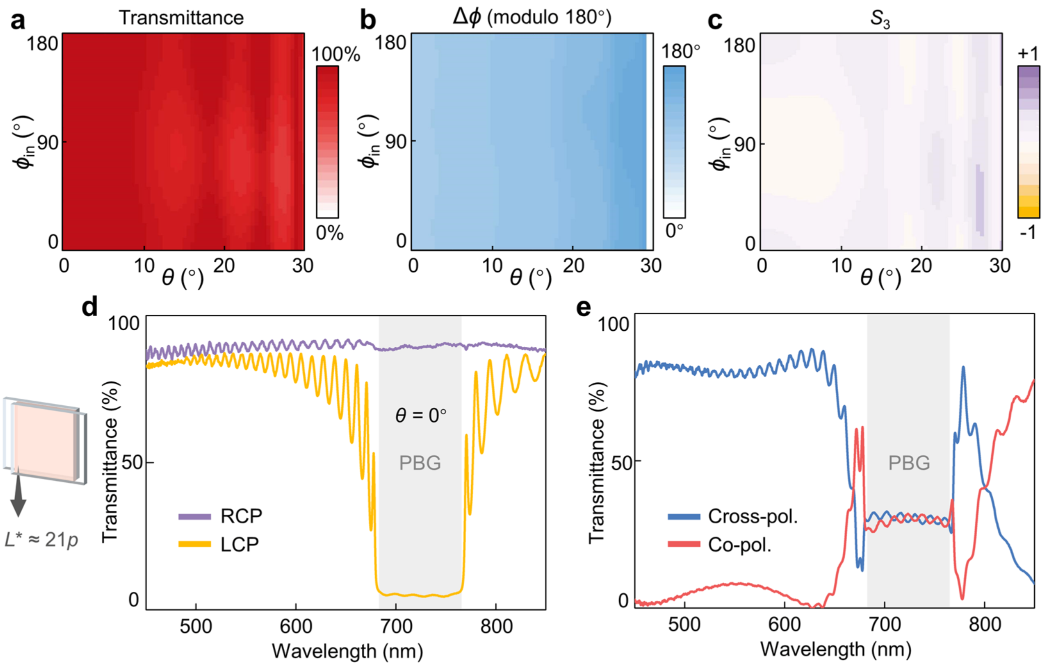

Fig. S13 | Optical properties of the CLC logic gate with **short interaction length** (L^* = 9.5 μm). **a-c**, Simulated results of (**a**) the transmittance without analyzer, (**b**) polarization rotation $\Delta\phi$, and (**c**) S_3 as a function of θ and the input polarization direction ϕ_{in}, for the operating wavelength 632.8 nm. **d**, Measured transmittance spectra for normal incidence with RCP (purple) and LCP (yellow), respectively. **e**, Measured co-polarized (red) and cross-polarized (blue) transmittance spectrum for normal incidence with ϕ_{in} = 90°. The schematic of the 9.5-μm-thick CLC device is illustrated on the left. In this case, $L^* \approx 21p$, lower than the presented $L \approx 155p$ by an order of magnitude. Compared to that of Fig. 3b, only few transmittance extremes can be observed here, which implies that the polarization rotation range is quite limited.

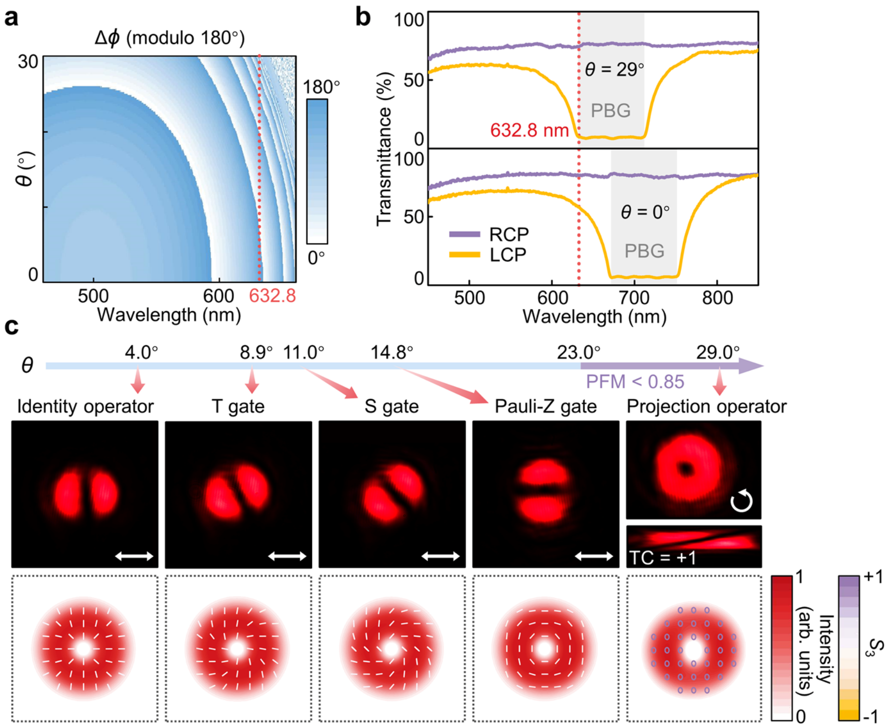

Fig. S14 | Optical properties of the CLC logic gate when **the operating wavelength is closer to the short-wavelength edge of PBG** (λ = 632.8 nm and $n_o p$ = 670 nm). **a**, Simulated dependence of $\Delta\phi$ on the wavelength and θ for ϕ_{in} = 0°, with target λ labelled by red dotted line. **b**, Measured transmittance spectra of the CLC device under RCP (purple) and LCP (yellow) incidence at various θ. The PBG and λ are labelled. **c**, For the radially polarized input state, captured analyzed intensity profiles, and simulated polarization maps of the output states when the CLC device performs an identity operator, a T gate, an S gate, a Pauli-Z gate, and a projective operator, corresponding to θ = 4.0°, 8.9°, 11.0°, 14.8°, and 29.0°, respectively. White arrows denote horizontal analyzers. Polarization maps are shown as false-color intensity and polarization ellipses colored according to S_3. Compared to Fig. 2,5, $\lambda/(n_o p)$ is bigger here, implying stronger dispersion of the optical rotatory power near the operating wavelength. Therefore, the logical rotation depends more sensitively on θ. Meanwhile, PFM < 0.85 for θ > 23.0°, and the PBG already covers 632.8 nm when θ = 29.0°, indicating a narrower dynamic range of θ.

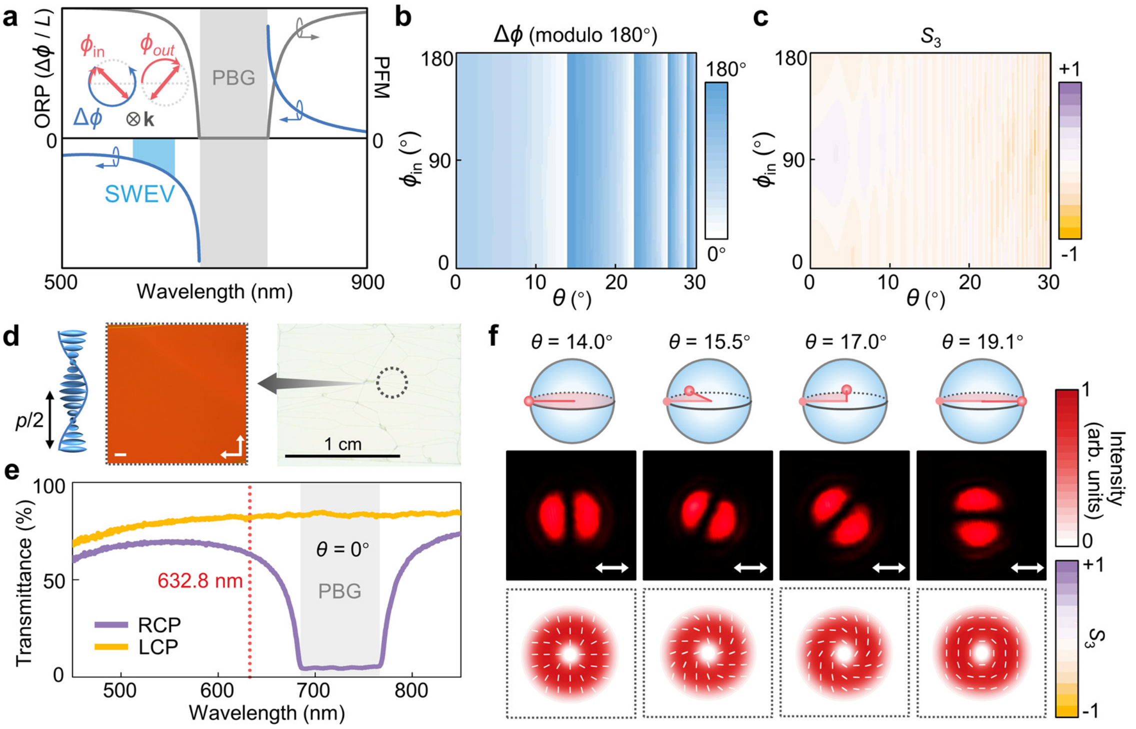

Fig. S15 | Optical properties of the CLC logic gate with **the opposite chirality** (right-handed). **a**, Analytical optical rotatory power (ORP) and the polarization figure of merit (PFM) as functions of the wavelength for linearly polarized normal incidence. SWEV, short-wavelength edge vicinity; L, interaction length. Inset: the input (ϕ_{in}) and output (ϕ_{out}) linear polarization direction, and the polarization rotation ($\Delta\phi$). **k** denotes the wave vector. **b-c**, Simulated dependences of (**b**) the polarization rotation $\Delta\phi$, and (**c**) the Stokes parameter S_3 on θ ranging from 0° to 30° and ϕ_{in} ranging from 0° to 180°. **d**, Schematic of the right-handed chiral superstructures with the helical pitch p, the cross-polarized reflective optical micrograph of the region indicated by a black dotted circle, and the macroscopic photograph of the CLC device. The scale bars of the micrograph and the macrograph are 100 μm and 1 cm, respectively. **e**, Measured transmittance spectra for normal incidence with RCP (purple) and LCP (yellow), respectively. The PBG and the operating wavelength 632.8 nm are labelled. **f**, For the radially polarized input state, captured analyzed intensity profiles and simulated polarization maps of the output states with the logical rotation angle Φ = 0, -π/4, -π/2, and -π, corresponding to θ = 14.0°, 15.5°, 17.0°, and 19.1°, respectively. White arrows denote horizontal analyzers. Polarization maps are shown as false-color intensity and polarization ellipses colored according to S_3. Compared to Fig. 2-5, the right-handed CLC logic gate introduces a negative value of the logical rotation angle of non-separable state.

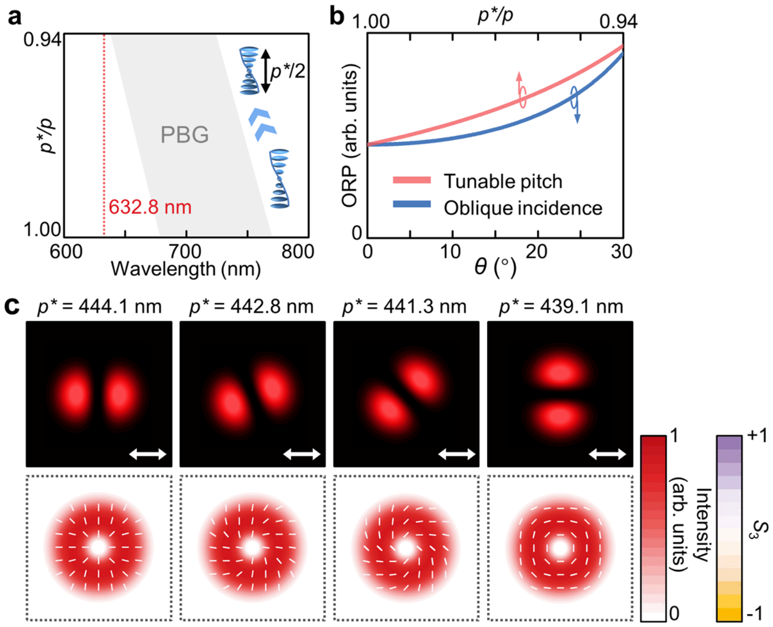

Fig. S16 | The dynamic logical rotation via CLC chiral superstructures with tunable helical pitch. **a**, Analytical dependence of the PBG on the variable pitch p^* under normal incidence. The original pitch is set as $p = 450$ nm. The operating wavelength $\lambda = 632.8$ nm is labelled. Inset: schematic of the chiral superstructures with shortened pitch. **b**, Analytical optical rotatory power (ORP) as a function of θ ranging from 0° to 30° (blue), and p^* ranging from $1.00p$ to $0.94p$ (red), respectively. The analytical theory is described in Supplementary Note 2. **c**, For the input radially polarized state, simulated analyzed intensity profiles and polarization maps of the output states when the CLC device performs an identity operator, a T gate, an S gate, and a Pauli-Z gate, corresponding to p^* = 444.1 nm, 442.8 nm, 441.3 nm, and 439.1 nm, respectively. The above results imply the feasibility of controllable logical rotation via stimuli-responsive CLC materials, for example, driven by light[34] or heat[35].

Supplementary Information References

1 Forbes, A., de Oliveira, M. & Dennis, M. R. Structured light. *Nat. Photon.* **15**, 253–262 (2021).

2 He, C., Shen, Y., Forbes, A. Towards higher-dimensional structured light. *Light Sci. Appl.* **11**, 205 (2022).

3 Spreeuw, R. J. C. Classical wave-optics analogy of quantum-information processing. *Phys. Rev. A* **63**, 062302 (2001).

4 Aiello, A. et al. Quantum−like nonseparable structures in optical beams. *New J. Phys.* **17**, 043024 (2015).

5 Korolkova, N. & Leuchs, G. Quantum correlations in separable multi-mode states and in classically entangled light. *Rep. Prog. Phys.* **82**, 056001 (2019).

6 Shen, Y. & Rosales-Guzmán, C. Nonseparable states of light: from quantum to classical. *Laser Photon. Rev.* **16**, 2100533 (2022).

7 Zhan, Q. Cylindrical vector beams: from mathematical concepts to applications. *Adv. Opt. Photon.* **1**, 1–57 (2009).

8 Nape, I. et al. Revealing the invariance of vectorial structured light in complex media. *Nat. Photon.* **16**, 538–546 (2022).

9 Allen, L., Beijersbergen, M. W., Spreeuw, R. J. C. & Woerdman, J. P. Orbital angular momentum of light and the transformation of Laguerre-Gaussian laser modes. *Phys. Rev. A* **45**, 8185-8189 (1992).

10 Fang, X. et al. Nanophotonic manipulation of optical angular momentum for high-dimensional information optics. *Adv. Opt. Photon.* **13**, 772-833 (2021).

11 Milione, G., Sztul, H. I., Nolan, D. A. & Alfano, R. R. Higher-order Poincaré sphere, Stokes parameters, and the angular momentum of light. *Phys. Rev. Lett.* **107**, 053601 (2011).

12 Faryad, M. & Lakhtakia, A. The circular Bragg phenomenon. *Adv. Opt. Photon.* **6**, 225-292 (2014).

13 Belyakov, V. A. *Diffraction Optics of Complex-Structured Periodic Media* (Springer Nature, 2019).

14 Dreher, R. & Meier, G. Optical properties of cholesteric liquid crystals. *Phys. Rev. A* **8**, 1616-1623 (1973).

15 Oldano, C. Dispersion relation for propagation of light in cholesteric liquid crystals. *Phys. Rev. A* **27**, 3291-3299 (1983).

16 Chen, C. W. & Khoo, I. C. Optical vector field rotation and switching with near-unity transmission by fully developed chiral photonic crystals. *Proc. Natl. Acad. Sci. U. S. A.* **118**, e2021304118 (2021).

17 Khoo, I. C. *Liquid Crystals* (Wiley, 2022).

18 Sterling, T. H. & Hayes, C. F. Optical rotatory power of a cholesteric liquid crystal for obliquely incident light using multiple scaling. *Mol. Cryst. Liq. Cryst.* **43**, 279-286 (1977).

19 Berreman, D. W. & Scheffer, T. J. Reflection and transmission by single-domain cholesteric liquid crystal films: theory and verification. *Mol. Cryst. Liq. Cryst.* **11**, 395-405 (1970).

20 Chen, C.-W., Feng, T.-M., Wu, C.-W., Lin, T.-H. & Khoo, I. C. Massive, soft, and tunable chiral photonic crystals for optical polarization manipulation and pulse modulation. *Appl. Phys. Rev.* **10**, 011413 (2023).

21 Nielsen, M. A. & Chuang, I. L. *Quantum computation and quantum information* (Cambridge University Press, 2010).

22 Zhou, C. H. & Liu, L. R. Numerical study of Dammann array illuminators. *Appl. Opt.* **34**, 5961-5969 (1995).

23 Jaeger, R. C. & Blalock, T. N. *Microelectronic circuit design* (McGraw-Hill, 2010).

24 Postler, L. et al. Demonstration of fault-tolerant universal quantum gate operations. *Nature* **605**, 675-680 (2022).

25 Jiang, Z. H. et al. A single noninterleaved metasurface for high-capacity and flexible mode multiplexing of higher-order Poincaré sphere beams. *Adv. Mater.* **32**, 1903983 (2020).

26 Qian, C. et al. Performing optical logic operations by a diffractive neural network. *Light Sci. Appl.* **9**, 59 (2020).

27 Chen, S.-Q. et al. Cylindrical vector beam multiplexer/demultiplexer using off-axis polarization control. *Light Sci. Appl.* **10**, 222 (2021).

28 Kim, J., Miskiewicz, M. N., Serati, S. & Escuti, M. J. Nonmechanical laser beam steering based on polymer

polarization gratings: design optimization and demonstration. *J. Lightwave Technol.* **33**, 2068-2077 (2015).

29 Dorrah, A. H. & Capasso, F. Tunable structured light with flat optics. *Science* **376**, eabi6860 (2022).

30 Shastri, K. & Monticone, F. Nonlocal flat optics. *Nat. Photon.* **17**, 36-47 (2023).

31 D'errico, A. et al. Two-dimensional topological quantum walks in the momentum space of structured light. *Optica* **7**, 108-114 (2020).

32 Di Colandrea, F. et al. Ultra-long quantum walks via spin–orbit photonics. *Optica* **10**, 324-331 (2023).

33 Meng, X.-Y. et al. Compact optical convolution processing unit based on multimode interference. *Nat. Commun.* **14**, 3000 (2023).

34 Zheng, Z. et al. Digital photoprogramming of liquid-crystal superstructures featuring intrinsic chiral photoswitches. *Nat. Photon.* **16**, 226–234 (2022).

35 Zhang. Y.-H. et al. Dynamically selective and simultaneous detection of spin and orbital angular momenta of light with thermoresponsive self-assembled chiral superstructures. *ACS Photon.* **9**, 1050-1057 (2022).
